# Quatsomes Formulated with l-Prolinol-Derived Surfactants as Antibacterial Nanocarriers of (+)-Usnic Acid with Antioxidant Activity

**DOI:** 10.1021/acsanm.1c04365

**Published:** 2022-05-09

**Authors:** Sara Battista, Mariana Köber, Pierangelo Bellio, Giuseppe Celenza, Luciano Galantini, Guillem Vargas-Nadal, Lorenza Fagnani, Jaume Veciana, Nora Ventosa, Luisa Giansanti

**Affiliations:** † Dipartimento di Scienze Fisiche e Chimiche, Università degli Studi dell’Aquila, Via Vetoio, 67010 Coppito, L’Aquila, Italy; ‡ Institut de Ciència de Materials de Barcelona (ICMAB-CSIC), Esfera Universitat Autónoma de Barcelona (UAB); Campus UAB s/n, E-08193 Cerdanyola del Vallès, Spain; § Dipartimento di Scienze Cliniche Applicate e Biotecnologie, Università degli Studi dell’Aquila, Via Vetoio, 67010 Coppito, L’Aquila, Italy; ∥ Dipartimento di Chimica, Università di Roma “Sapienza”, Piazzale Aldo Moro 5, 00185 Roma, Italy; ⊥ Networking Research Center on Bioengineering, Biomaterials and Nanomedicine (CIBER-BBN), Campus Universitari de Bellaterra, E-08193 Cerdanyola, Spain

**Keywords:** antibacterial activity, nanovesicles, antioxidant activity, depressurization of an expanded liquid organic solution-suspension, l-prolinol-derived surfactants, quatsomes, chemical structure-activity relationship, (+)-usnic acid

## Abstract

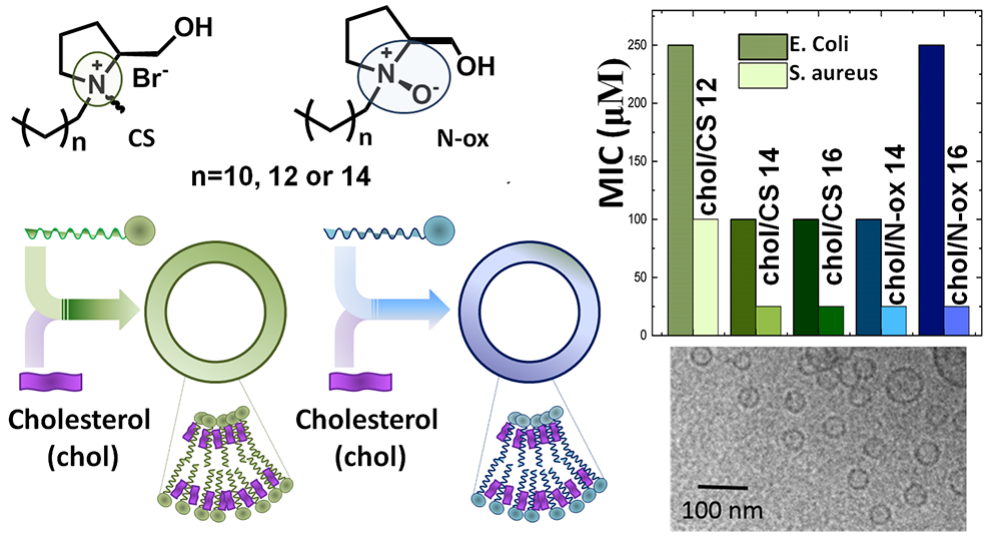

The efficacy of the treatment of bacterial infection is seriously reduced because of antibiotic resistance; thus, therapeutic solutions against drug-resistant microbes are necessary. Nanoparticle-based solutions are particularly promising for meeting this challenge because they can offer intrinsic antimicrobial activity and sustained drug release at the target site. Herein, we present a newly developed nanovesicle system of the quatsome family, composed of l-prolinol-derived surfactants and cholesterol, which has noticeable antibacterial activity even on Gram-negative strains, demonstrating great potential for the treatment of bacterial infections. We optimized the vesicle stability and antibacterial activity by tuning the surfactant chain length and headgroup charge (cationic or zwitterionic) and show that these quatsomes can furthermore serve as nanocarriers of pharmaceutical actives, demonstrated here by the encapsulation of (+)-usnic acid, a natural substance with many pharmacological properties.

## Introduction

Quatsomes are nanometric self-assembled aggregates composed of cholesterol (chol) and surfactants bearing a single chain and a quaternary ammonium moiety, which individually form crystals and micelles in aqueous solutions. Quatsomes have great potential in biomedical applications because they share some important features with liposomes but overcome some problems such as a lack of stability and aggregation over time. In fact, quatsomes are very stable and homogeneous in lamellarity and size, features that do not change in a wide range of temperatures or dilutions.^1,2^ They can be prepared by exploiting sonication or protocols that encompass the use of compressed fluids.^3^ As a consequence of their unique characteristics, these nanovesicles have gained increasing attention because they can be employed in a wide variety of applications like biological imaging^4−6^ and as drug-delivery systems.^3,7^ In fact, they can integrate small drugs or large biomolecules in the bilayer and/or in the aqueous core and their surface can be decorated with functional groups.^8^ Moreover, quatsomes themselves (devoid of any active principle) containing cetylpyridinium chloride showed antibacterial activity on a *Staphylococcus aureus* biofilm.^9^

**Chart 1:**
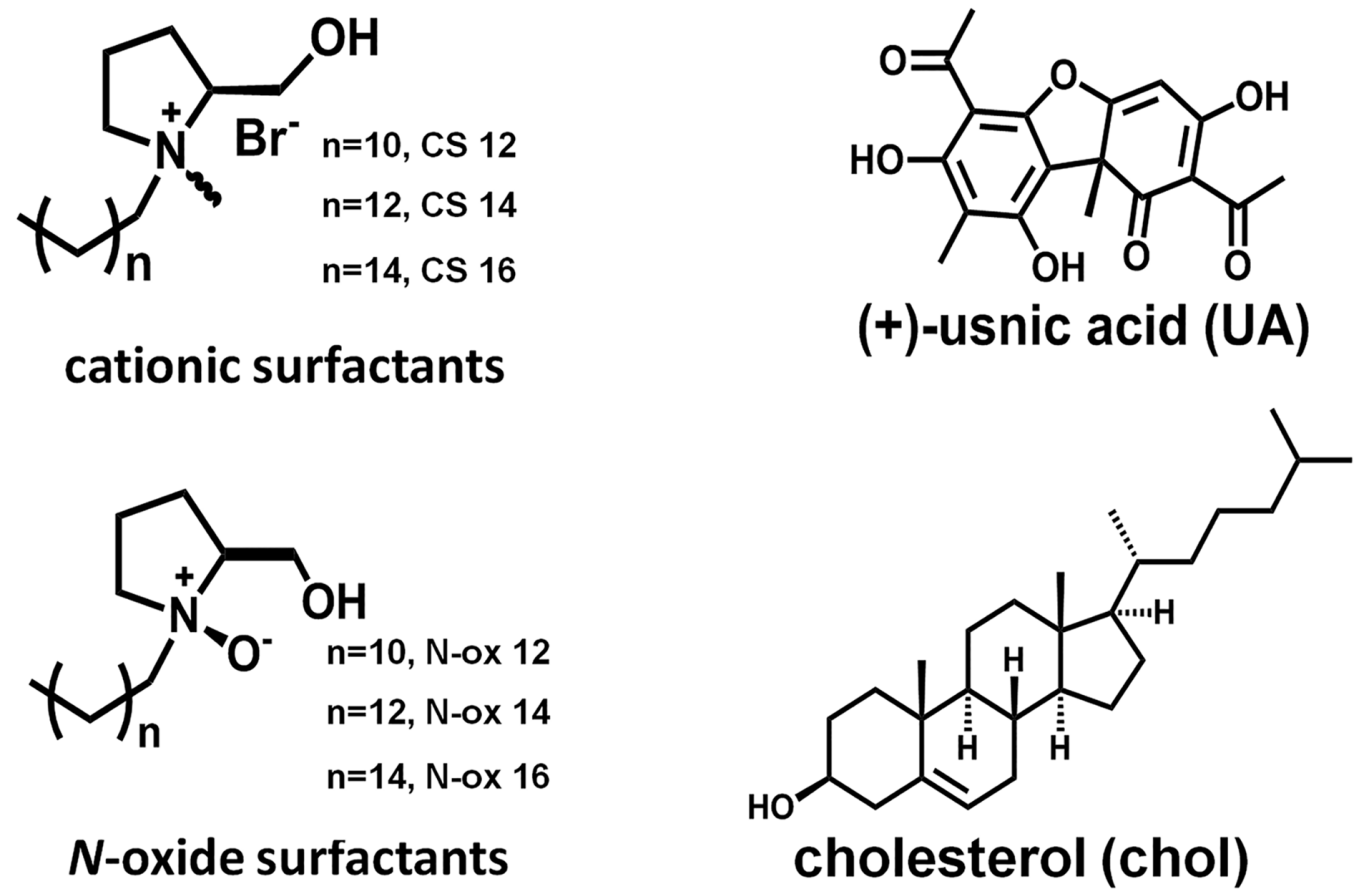
Quatsome Components and UA

Here we report on the preparation of quatsomes containing equimolar amounts of chol and one of the synthetic surfactants reported in Chart 1: three cationic and three *N*-oxide surfactants, with alkyl chain lengths of 12, 14, or 16 methylenes, respectively (CS **12**, CS **14**, CS **16**, N-ox **12**, N-ox **14**, and N-ox **16**). These surfactants are *l*-prolinol-derived surfactants, which were shown to enhance the efficacy of DNA and other lipid drug-delivery systems,^10−12^ reduce the effective dose of some antibiotics^13^ and confer antibacterial activity to liposomal formulations.^14^ The possibility of including these surfactants at high molar percentage in the formulations can maximize their pharmacological potential. We exploited depressurization of an expanded liquid organic solution–suspension (DELOS-SUSP), a green and scalable methodology that allows one to work in sterile conditions.^3^ With this one-step technique, it is possible to obtain unilamellar nanoscale quatsomes showing narrow size distribution and more homogeneous composition compared to those obtained with conventional vesicles preparation protocols.^15,16^ It consists of depressurization of a CO_2_-expanded solution containing chol, dissolved in acetone or ethanol, into an aqueous phase containing the quaternary ammonium surfactant. The use of supercritical fluids grants easier control of the morphology and the dimensions of the nanovesicles in order to achieve high reproducibility.^1^ (+)-Usnic acid (UA; Chart 1), a pharmacologically active (antibacterial,^17^ antiproliferative,^18^ antifungean,^19^ antiviral,^20^ and antiinflammatory^21^) substance produced by many lichens, was also loaded into the formulations. Unfortunately, UA is scarcely soluble in water^21^ and shows dose-dependent hepatotoxicity.^22^ As a consequence, its application has been limited to topical ointments, oral care products or cosmetic formulations.^23−25^ It is clear that the inclusion of UA in drug-delivery systems would take advantage of its several pharmacological properties upon systemic administration. For these reasons, we evaluated the entrapment efficiency (E.E.) of UA and its antibacterial activity when included in quatsomes. In particular, the antibacterial effectiveness of these nanovesicles, including or not UA, was evaluated on Gram-positive and Gram-negative bacteria and fungal strains. In a previous investigation, liposomes containing analogous structurally related *l*-prolinol derivatives showed a good ability to efficiently deliver UA to *S. aureus* bacterial cells.^10^ Because the biological activity of UA in some cases can be related to the antioxidant one,^26^ we assessed also the antioxidant effectiveness of quatsomes/UA. In fact, we previously showed that this property can significantly vary as a function of the composition of the systems in which it is included.^27−31^ The same evaluation was carried out in liposomes prepared either with thin-film hydration (TFH)^32^ or with DELOS-SUSP^33^ and formulated with 1,2-dimyristoyl-*sn*-glycero-3-phosphocholine, chol, and 10 mol % of the same synthetic surfactants as those used in this investigation. The aim of this study was to correlate the physicochemical properties of the aggregates with their biological effects to individuate which factors mostly affect the ability of the formulations to interact with the biological *milieu* and to optimize their potential in terms of drug-delivery efficacy.

## Experimental Section

### Instrumentation

Quatsomes were prepared using DELOS-SUSP equipment. The size and ζ potential of quatsomes were measured by dynamic light scattering (DLS) using the apparatus described in ref (32). The water used was pretreated with a Milli-Q Advantage A10 water purification system (Millipore Ibérica, Madrid, Spain). UV measurements were carried out on a Cary 50 UV–vis double-beam spectrophotometer (Varian) and on the microplate reader iMark (BioRad, Milan, Italy). Cryogenic transmission electron microscopy (cryo-TEM) was performed using the apparatus described in ref (33).

### Materials

Chol (purity 95%) was purchased from Panreac (Barcelona, Spain). Milli-Q water (Millipore Ibérica, Madrid, Spain), ethanol (EtOH; Teknocroma, Sant Cugat del Vallès, Spain), and dimethyl sulfoxide (DMSO; Sigma-Aldrich, Milan, Italy) in high purity were the solvents used for the preparation of the samples by DELOS-SUSP equipment. Carbon dioxide (CO_2_; 99.9% purity) was purchased from Carburos Metálicos S.A. (Barcelona, Spain). Sabouraud dextrose broth, Sabouraud dextrose agar (Liofilchem), and Mueller–Hinton broth (Biolife) were the media used for biological tests. UA, RPMI-1640 with l-glutamine, phenol red powder dissolved in 3-(*N*-morpholino)propanesulfonic acid at a final concentration of 0.165 M and pH 7.0 supplemented with 2% glucose (RPMI-1640 G2), dialysis tubing cellulose membrane (cutoff = 14000), CH_3_COONa, H_2_O_2_, and 2,2′-azinobis(3-ethylbenzothiazoline-6-sulfonic acid) diammonium salt (ABTS) were purchased from Sigma-Aldrich (Milan, Italy). Cationic surfactants (CSs; CS **12**, CS **14**, and CS **16**) and their corresponding *N*-oxides (N-oxs: N-ox **12**, N-ox **14**, and N-ox **16**) were prepared as reported in ref (27). All solvents and chemicals for the synthesis were used as purchased without further purification.

Methicillin-resistant *S. aureus* reference strain from the American Type Culture Collection (ATCC 43300), *Escherichia coli* (ATCC 25922), and *Candida albicans* (ATCC 64124) fungal strain were used as control organisms.

### Methods

#### Preparation of Quatsomes by DELOS-SUSP

The investigated quatsomes were prepared by DELOS-SUSP, a methodology based on the use of compressed fluids.^8^ Chol (4.87 mg) was dissolved in 1.2 mL of EtOH under the conditions described in ref (33). The organic phase was depressurized over 24 mL of prewarmed (*T*_w_) Milli-Q water containing around 5 mg of one of the CSs or N-oxs [molar ratio chol/CS (N-ox) = 1:1; total concentration of membrane components = 1 mM). During depressurization, N_2_ (11.5 MPa) was used to flush the reactor to achieve a constant *P*_w_. Quatsome suspensions were purified through a dialysis process to eliminate EtOH by exchanging the external medium, which was Milli-Q water at 25-fold the quatsome dispersion volume, four times within 1 h.

#### UA Loading in Quatsomes and E.E. Evaluation

UA loading in the DELOS-SUSP process: chol (4.87 mg) was dissolved in 1.15 mL of EtOH at working temperature *T*_w_. The proper amount of UA to obtain a final UA concentration equal to 5 × 10^–5^ M in DMSO (50 μL) was preheated at *T*_w_ and adjoined dropwise to the organic phase, which was then added to the high-pressure vessel. The molar ratio UA/quatsome components (*i.e.*, surfactant + chol) is 1:20. The remaining part of the protocol was the same as that described above (without UA). Quatsomes were treated with dialysis to eliminate DMSO and unentrapped UA, besides EtOH.

UA loading by incubation: a few microliters of a solution of 37.5 mM UA dissolved in DMSO was adjoined to previously prepared quatsomes to obtain a final molar ratio (quatsome components, *i.e.*, lipid + chol)/UA of 20:1; then the dispersion was warmed for 1 h at 40 °C. Quatsomes were treated with dialysis to eliminate the organic solvents (DMSO and EtOH) and UA not included in the aggregates.

E.E. of UA was assessed by comparing the intensity of its absorbance at 290 nm before and after the removal of free UA by dialysis.

#### DLS and ζ-Potential Measurements

Size and ζ-potential measurements were carried out at 25 °C on 1 mM quatsome solutions, soon after their preparation, after dialysis, and over time (up to 6 months) as described previously.^32^ We carried out measurements soon after quatsomes preparation to assess their formation. After 1 week, we repeated the measurements because it is known that quatsomes components rearrange in the bilayer in a stable organization over this period.

#### Cryo-TEM Measurements

The quatsomes morphology was studied by cryo-TEM measurements as described in ref (33).

#### Preparation of an ABTS^•+^ Reagent Solution

The solution containing the radical cation was prepared as reported in ref (33).

#### Evaluation of the Antioxidant Activity of Free or Loaded UA by ABTS^•+^ Methodology

A volume of 25 μL of ABTS^•+^ solution was rapidly added to 2450 μL of acetate buffer at pH 5.5 prepared as described above containing 1.38 × 10^–6^ M of free or quatsome-loaded UA (total water volume = 250 μL; final concentration in the cuvette of ABTS^•+^ = 9.17 × 10^–5^ M). Variation of the maximal absorbance at 417 nm was followed for 1 h. The reported results are the average of at least three repeated measurements. The absorbance decay over time was fitted using Origin Pro 2012 to quantify the contribution of UA to the degradation of ABTS^•+^, as described in refs (32) and (33) (τ_ABTS_ = 34.4 min).

#### Evaluation of the Antimicrobial/Antifungal Activity of Quatsome Formulations

The antimicrobial susceptibility of methicillin-resistant *S. aureus* and *E. coli* strains to quatsomes with or without UA was evaluated following the CLSI guidelines.^35^ Briefly, for the antibacterial susceptibility, 100 μL of a bacterial suspension in a 0.9% saline solution (NaCl) at a concentration of 5 × 10^–5^ CFU/mL was added to the wells of a 96-well microtiter plate containing 100 μL of 2-fold serially diluted free UA and quatsomes loaded with UA (with both loading methodologies) in cation-adjusted Mueller–Hinton broth. The experiments were then carried out as reported in ref (10).

The antimycotic effect of quatsomes with or without UA against *C. albicans* ATCC 64124 was determined in accordance with EUCAST guidelines (Edef 7.3.2).^36^ Briefly, a glycerol stock solution of yeast was inoculated into Sabouraud dextrose broth and subsequently subcultured onto Sabouraud dextrose agar. The inoculum at 0.5 McFarland in 0.9% saline solution NaCl was prepared by picking some colonies and following the method described in the EUCAST guidelines in order to reach the final concentration of (2.5–0.5) × 10^5^ CFU/mL. A total of 100 μL of inoculum was added to the wells of a 96-well flat bottom microtiter plate containing 100 μL of 2-fold serially diluted free UA and quatsomes loaded with UA in RPMI-1640 G2. The antifungal activity was determined by a microdilution method in 96-well microplates statically incubated for 24 h at 35 °C. Microorganism growth was spectrophotometrically quantified at 595 nm by a microplate reader. The minimum inhibitory concentration (MIC) for free UA and quatsomes loaded with UA was defined as the concentration of drug that decreases growth by 80% compared with that of organisms grown in the absence of drug. The MICs of quatsomes were indicated through nominal surfactant concentrations that were used for quatsome production.

## Results and Discussion

### Plain Quatsomes: Size, Morphology, and ζ Potential

**Figure 1:**
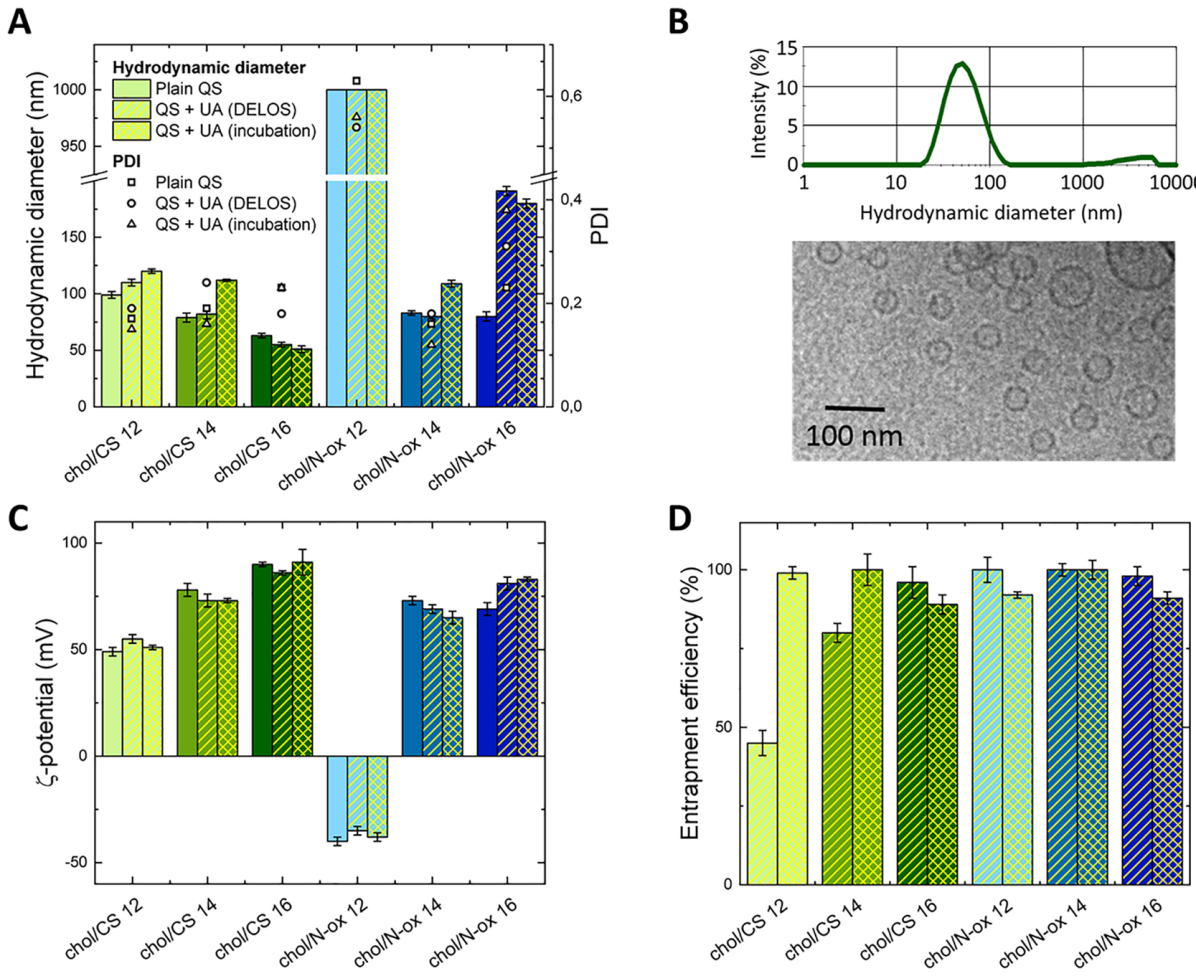
(A) Hydrodynamic diameter and PDI of nondialyzed quatsomes, devoid of UA or containing UA added in the quatsome formation process (DELOS) or to preformed quatsomes (incubation). Reported values were obtained 1 week after the quatsome preparation and correspond to the average of three independent measurements. The error bars indicate the standard deviation. (B) Cryo-TEM image and size distribution of chol/CS **16** quatsomes obtained by DLS. (C) ζ potential of the investigated plain and UA-containing quatsomes in water. (D) E.E. of UA loaded in the investigated quatsomes during vesicles formation. Graphs A, C, and D share the same legend: bars without lines (when present) indicate plain quatsomes, bars filled with yellow diagonal lines indicates quatsomes in which UA was directly added to the vessel and bars filled with yellow crossed lines indicate samples in which UA was included in the aggregates by incubation on preformed quatsomes, as reported in the legend of Figure 1A.

The dimensions and dispersity of quatsomes were measured immediately after their preparation, after 1 week (Figure 1A and Table S1) and over time up to 4 months (Table S2). The formulations differ in their surfactant chain lengths (12, 14, and 16 carbons) and/or headgroup charge (cationic, CSs, or zwitterionic, N-oxs). In the first weeks after preparation, all of the investigated formulations, except chol/N-ox **12**, yielded monomodal distributions of sizes with an average hydrodynamic diameter of 50–100 nm and low dispersities (polydispersity index, PDI, ≈ 0.2), obtained from cumulant analysis (Figure 1A and Table S1). Only chol/N-ox **12** yielded dimensions in the micrometer range with increased dispersity. After 4 months, the dispersity remained low, and average diameters increased by about 30–40 nm (Table S2), with the exception of chol/CS **16**, which was stable in size for 4 months and showed a spherical unilamellar morphology after this time (Figure 1B). Thus, the optimal nanovesicle stability was achieved with a longer alkyl chain (C16), which grants increased hydrophobic and van der Waals interaction.

All formulations except chol/N-ox **12** exhibited a positive net surface charge, with ζ potentials of >50 mV 1 week after the quatsome preparation (Figure 1C and Table S3). Chol/N-ox **12** featured a positive potential around 50 mV soon after the preparation (similarly to CS **12** containing quatsomes) that turned negative after 1 week, indicating a loss of stability of the bilayer and thus confirming the low stability of this formulation. Reasonably, in the lipid rearrangement, there is a variation of the exposure of the polar headgroup that brings a higher association of counterions to the positively charged group of the *N*-oxide moiety with consequent reduction of the ζ potential of the formulations. Positive ζ potentials were observed for liposomes containing these zwitterionic N-ox surfactants, with the exception of N-ox **12**, independently of the methodology used for their preparation.^33,34^ Our data point out that, when quatsomes contain CS **12**, the nature of the polar headgroup is more relevant than that for longer alkyl chains, probably because the extent of hydrophobic and van der Waals interactions is significantly reduced. In fact, even if the alkyl chain contains only two or four methylenes less, the aggregation number of quatsomes is very high and the overall effect is reflected not only on their stability but also on the hydrophobic/hydrophilic balance of the aggregates. In particular, N-ox **12** forms the least stable quatsomes, indicating that the repulsive interactions among the zwitterionic surfactants, despite their overall electrical neutrality, are higher than those among the corresponding cationic CS **12**. Literature reports indicate that surfactants containing the *N*-oxide moiety and a C12 chain can destabilize the bilayer even at low molar percentage.^37,38^ Also liposomes prepared with DELOS-SUSP containing CS **12** and N-ox **12** were less stable than the others, even if their destabilizing effect was less evident,^34^ likely because of the lower molar fraction of the surfactant in the total composition (10 mol % in liposomes *vs* 50 mol % in quatsomes). Liposomes with the same composition prepared according to TFH were considerably less stable,^33^ even more than quatsomes that contain a 5-fold amount of synthetic surfactant. Even if N-ox **12** and the other surfactants with longer alkyl chains differ by only two or four methylenes, it is not so unusual that this difference can be crucial in determining properties of the aggregates that form or in which they are included,^33,34,37,38^ Also micelles formed by the same synthetic surfactants used in this investigation show different properties as a function of the chain length.^27^ Moreover, many literature reports on aggregates containing or formulated with surfactants with unrelated molecular structure from the investigated ones but differing for only two or four methylenes in the hydrophobic chain demonstrate that this parameter can play a pivotal role in determining the properties of the aggregates.^39−43^ As a whole, the obtained results confirm the following: (i) the high stability of quatsomes containing surfactants bearing at least 14 carbon atoms in the chain (even in this case in which cationic or zwitterionic synthetic surfactants were used for the first time for their preparation); (ii) the high stability and homogeneity of the bilayer of the aggregates prepared using DELOS methodology; (iii) the pivotal role of the hydrophobic/hydrophilic balance in determining the nanovesicle properties.

### UA-Containing Quatsomes: Size, ζ Potential, and E.E

The inclusion of UA in the formulations barely affected their size or ζ potential, regardless of whether UA was added during vesicle formation or by incubation (Figure 1A and Table S1 and also Figure 1C and Table S3). The dialysis (carried out 1 week after their preparation) did not affect the dimensions of the nanovesicles except for CS **12** quatsomes, which showed, like the N-ox **12** ones, an increase of the dimensions and PDI value, together with incipient precipitation (data not shown). Different from liposomes,^32,33^ for quatsomes the high amount of synthetic surfactant (50 mol %) seems to mask the effect of UA on the ζ potential. Moreover, the absence of the phospholipid (that bears two long chains) could make the bilayer slacker with respect to liposomes, allowing UA to more deeply penetrate the hydrophobic region. In fact, it is well-known that the contribution to membrane mechanics in a bilayer is strictly dependent on the chemical structure of the lipid/lipids that compose it.^44^ All of the samples mainly showed a slight decrease (about 10 mV) of ζ potential over time, more prominent (30 mV) in the case of CS **12** quatsomes probably because of their lower stability which leads to lipid rearrangement. A similar decrease of the potential for the sample containing CS **12** was observed upon dialysis 1 week after the preparation, thus confirming the low stability of the formulation.

All of the formulations showed very high E.E. (Figure 1D and Table S4), indicating that UA can penetrate the bilayer and its presence does not disturb lipid organization. The only exception was the formulation containing CS **12**, in which UA was included in the vessel during formation of the vesicles: in this case, the E.E. was around 50%. In general, this formulation showed a different behavior over time and when subjected to dialysis with respect to the samples containing CSs with longer chains. Moreover, the high E.E. observed through incubation of UA suggests a different location of this molecule in the bilayer or a different permeability depending on the methodology of the preparation.

An interesting result is that the formulation containing N-ox **12** showed a high E.E. (both by the addition of UA in the reactor and by incubation) even if the ζ potential in the last case was negative. It is possible that the low stability of this sample implies a reduction of lipid packing and thus UA can more easily penetrate the bilayer.

### Evaluation of the Antioxidant Activity of Free or Loaded UA by ABTS^•+^ Methodology

**Figure 2:**
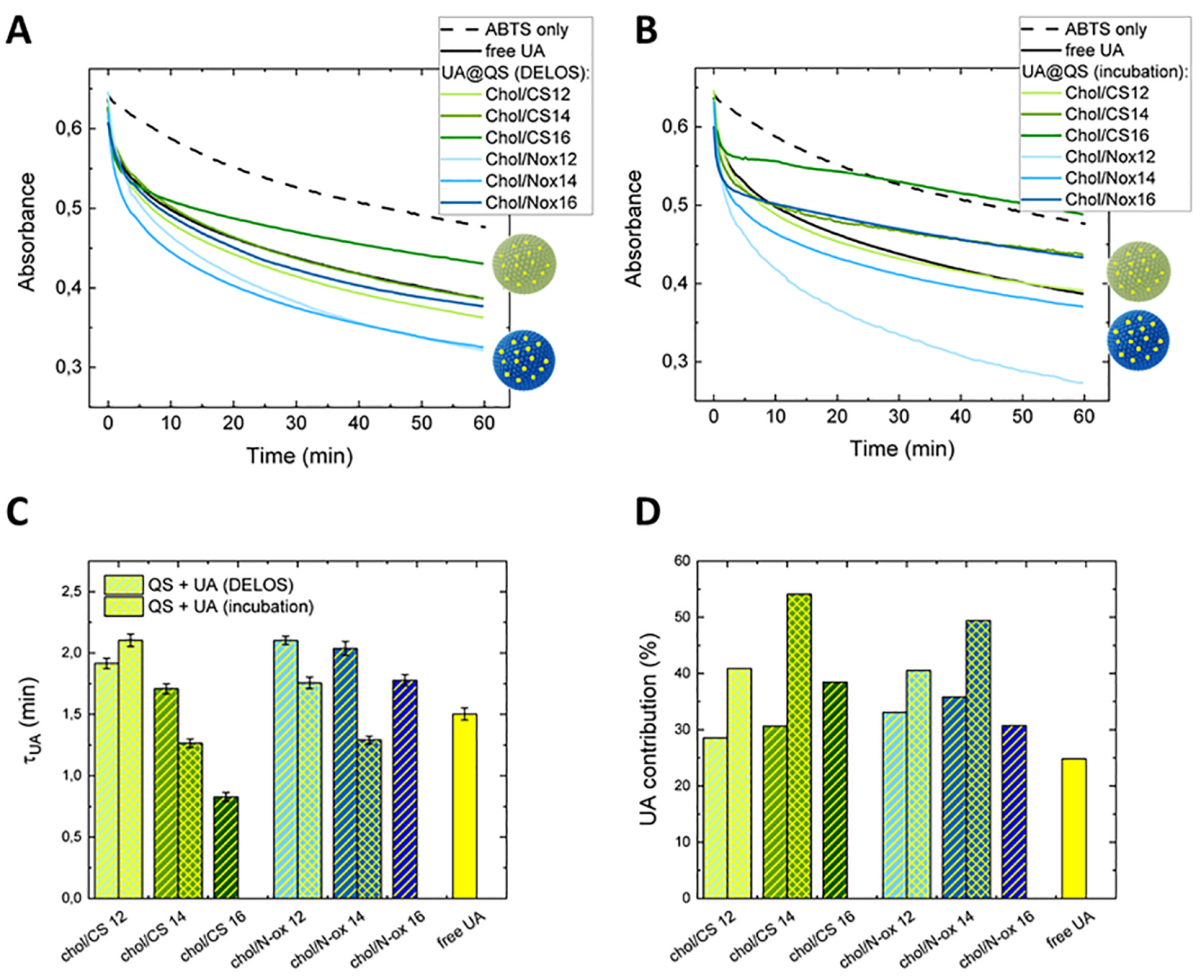
ABTS^•+^ degradation kinetics determined through absorption measurements at 417 nm over time, in the presence (solid black line) or absence (dashed black line) of free UA (*i.e.* UA added in solution and not included in quatsomes), as well as UA loaded into the different quatsome formulations: (A) UA incubated with preformed quatsomes; (B) UA loaded into the DELOS process, *i.e.* present during quatsome formation. Characteristic time constant τ_UA_ (C) and total contribution (D) of UA to the degradation of ABTS^•+^. Comparison of free UA (yellow bar) with UA added to the quatsomes after vesicle preparation (bars filled with yellow diagonal lines) or during vesicle preparation (bars filled with yellow crossed lines). Standard errors of τ_UA_ shown in the graph are obtained from the fit. For *A*_UA_/*A*_tot_ (%), standard errors are smaller than 1%. Parts C and D share the same legend.

The antioxidant activity of natural compounds can significantly vary when they are embedded in a lipid bilayer,^45−49^ so we decided to assess the antioxidant activity of UA included in quatsomes according to a commonly used assay.^34^ Briefly, the radical cation of ABTS (obtained as described in the Experimental Section) was added to the quatsomes suspension, yielding a strong absorbance at 417 nm that vanishes upon its reduction to ABTS. The effect of an antioxidant compound on the reduction rate was evaluated following the reduction of the characteristic absorbance peak at 417 nm over time. The radical cation is extremely stable at low pH, whereas it is rapidly reduced in basic solutions, so pH 5.5 was chosen as a compromise that provides a low ABTS^•+^ degradation rate without affecting quatsomes stability. The slow degradation rate of ABTS^•+^ increased in the presence of a fixed amount of free or quatsome-included UA, as expected (Figure 2A,B), whereas quatsomes devoid of UA did not affect the ABTS^•+^ degradation rate (data not shown).

In general, formulations containing N-oxs showed higher antioxidant activity than those containing CSs. The activity was stronger for N-oxs with shorter chain lengths (12 and 14). Similar effects were observed independently of the preparation method of the UA-loaded quatsome formulations (Figure 2A,B). However, those obtained by UA incubation with preformed quatsomes led to a remarkable and more pronounced degradation of ABTS^•+^ in the presence of N-ox **12** probably because of a different organization and/or compaction of the bilayer. For the samples containing surfactants bearing a C16 chain, the trend was respected but complex phenomena occurred, especially if UA was incubated. The chain length and nature of the polar headgroup significantly influence the antioxidant activity of UA also when it is included in pure micelles formed by the same surfactants used in this study.^27^ In general, the polarity and rigidity of the microenvironment experienced by a compound play a crucial role in its exerted antioxidant activity.^50−52^ The same molecules in spherical or rodlike micelles of the same composition show different antioxidant activities as a function of their different locations inside the aggregates.^53^ The results that we obtained with UA can be due to the different ζ potentials of quatsomes and/or to the different locations of UA inside the aggregates. To better understand these aspects, we also fitted all of the curves with a double-exponential decay, considering the spontaneous and UA-related degradation of free ABTS^•+^. The time constant τ_UA_ of the UA-related degradation of free ABTS^•+^, reported in Figure 2C, clearly shows the impact of the quatsome formulation on the UA-related degradation kinetics. As observed in the preparation of liposomes by TFH,^32^ UA contributes about 30–40% to the process (Figure 2D), especially for incubated samples. Interestingly, in the case of liposomes prepared by DELOS,^33^ no effect was observed when the same experiments were carried out, indicating that in this case the high packing of lipids hampers the penetration of the ABTS radical cation in the bilayer. As a consequence, it cannot reach UA therein. It is thus confirmed that in quatsomes, despite the high stability of the bilayer, the absence of the phospholipid component (which bears two alkyl chains) reduces the hydrophobic and van der Waals interaction among lipids thus making the bilayer more permeable.

### Evaluation of the Antimicrobial/Antifungal Activity of Quatsome Formulations

The antibacterial activity of the different quatsome formulations (except the highly polydisperse chol/N-ox **12**) was evaluated on methicillin-resistant *S. aureus* (ATCC43300, Gram-positive) and *E. coli* (ATCC 25922, Gram-negative) bacterial strains (Table 1 and Figure S5) and on a *C. albicans* fungal strain. On *C. albicans* (ATCC 64124). We did not observe any effect for any of the investigated formulations except for chol/CS **16**, which showed a small growth reduction at a total lipid concentration of about 1 × 10^–3^ M (0.5 × 10^–3^ M of surfactant). On *S. aureus*, quatsome formulations containing surfactants bearing C14 or C16 chains showed a MIC of about 2.5 × 10^–5^ M (nominal surfactant concentration of 1.25 × 10^–5^ M), whereas samples containing CS **12** showed a MIC of about 1 × 10^–4^ M (0.5 × 10^–4^ M of surfactant). On *E. coli*, in the presence of surfactants CS **14**, CS **16** and N-ox **14**, the observed MIC was about 1 × 10^–4^ M (0.5 × 10^–4^ M of surfactant), whereas for the formulations chol/CS **12** and chol/N-ox **16**, it was about 2.5 × 10^–4^ M (1.25 × 10^–4^ M of surfactant). In general, the antibacterial activity of the formulations increases with the ζ potential, which is known to promote interaction of the aggregates with bacterial membranes.

**Table 1:** MIC Defined as the Concentration of the Different Quatsome Formulations That Reduces Growth by 80% Compared to Untreated Organisms on Methicillin-Resistant *S. aureus* ATCC43300 and *E. coli* ATCC 25922 Bacterial Strains (Molarity of Nominal Concentrations of Quatsome Components)

formulation	MIC *E. coli* (M)	MIC *S. aureus* (M)
chol/CS **12**	2.50 × 10^–4^	1.00 × 10^–4^
chol/CS **14**	1.00 × 10^–4^	2.50 × 10^–5^
chol/N-ox **14**	1.00 × 10^–4^	2.50 × 10^–5^
chol/CS **16**	1.00 × 10^–4^	2.50 × 10^–5^
chol/N-ox **16**	2.50 × 10^–4^	2.50 × 10^–5^

In all cases, the presence of UA in the formulations did not alter the MIC values independently from the methodology used for UA loading. The low UA concentrations at the MICs of these quatsomes ([UA] between 1.5 and 4.3 μg/mL, 4.4 and 12.5 μM, for *E. coli* and between 0.4 and 1.7 μg/mL, 1.2 and 5.0 μM, for *S. aureus*, much lower than the MIC value of free UA, which is 125 μg/mL, 360 μM, for *E. coli*(54) and between 1 and 8 μg/mL, 2.9 and 23 μM, for *S. aureus*(55)) make it difficult to appreciate any eventual synergistic effect, if present. In general, in the presence of the same formulations, we observed lower MIC values for the Gram-positive strain than for the Gram-negative one, normally very difficult to treat. Considering the MIC values of all of the formulations, expressed in micrograms per milliliter (Figure S5), for *S. aureus*, the MICs cover the range from 8.7 μg/mL (2.5 × 10^–5^ M chol/N-ox **14**) to 37.5 μg/mL (1 × 10^–4^ M chol/N-ox **12**) whereas for *E. coli*, the MICs are in the range of 35 μg/mL (1 × 10^–4^ M chol/N-ox **14**) to 93.8 μg/mL (2.5 × 10^–4^ M chol/CS **12**). Consequently, these formulations, although not antimicrobial molecules but supramolecular aggregates, have MIC values that are comparable to those of several antibiotics used in clinical practice.^35^

The results obtained by the biological experiments are encouraging because they highlight the relevant potential of the investigated formulations for antibacterial treatments. Further detailed investigations on the cytotoxicity of the formulations toward mammalian cells and specific studies to ascertain the mechanism of interaction will be carried out on the most promising formulations.

## Conclusions

Nanoscale quatsomes formulated with structurally related *l*-prolinol-derived surfactants were prepared and characterized. As a whole, the supramolecular nanometric structures containing longer chains (C14 and C16) were very stable and showed a good ability to entrap UA, demonstrating high potential as a drug-delivery system. UA included in quatsomes maintained its antioxidant activity, which was particularly strong for N-ox formulations bearing short chain lengths. Moreover, the investigated quatsomes showed good antibacterial activity even on Gram-negative bacteria, demonstrating that they are good candidates for the treatment of bacterial infections alone or in association with an active principle that they can load.

## Supporting Information Available

Additional data on the size and ζ potential of the investigated quatsomes and MIC reported in micrograms per milliliter of the total lipid concentrations (PDF)
