# Supplementary material for: Quatsomes Formulated with l-Prolinol-Derived Surfactants as Antibacterial Nanocarriers of (+)-Usnic Acid with Antioxidant Activity
Source: ACS Appl Nano Mater. 2022 May 9;5(5):6140–8. doi: 10.1021/acsanm.1c04365 (PMC9150064; doi:10.1021/acsanm.1c04365)
Supplement: Supplementary file 1 — an1c04365_si_001.pdf [file an1c04365_si_001.pdf]

Quatsomes formulated with L-Prolinol-Derived Surfactants as Antibacterial Nanocarriers of (+)-Usnic Acid with Antioxidant Activity

Sara Battista,[a] *Mariana Köber,*[b,e] *Pierangelo Bellio,*[c] *Giuseppe Celenza,*[c] *Luciano Galantini,*[d] *Guillem Vargas-Nadal,*[b] *Lorenza Fagnani,*[c] *Jaume Veciana,*[b,e] *Nora Ventosa,*[b,e*] *Luisa Giansanti*[a*]

[a]Dipartimento di Scienze Fisiche e Chimiche, Università degli Studi dell'Aquila, Via Vetoio, 67010 Coppito (AQ) (Italy)

[b]Institut de Ciència de Materials de Barcelona (ICMAB-CSIC), Esfera UAB; Campus UAB s/n; E-08193 Cerdanyola del Vallès, Spain

[c]Dipartimento di Scienze Cliniche Applicate e Biotecnologie, Università degli Studi dell'Aquila, Via Vetoio, 67010 Coppito (AQ) (Italy)

[d]Dipartimento di Chimica, Università di Roma "Sapienza", Piazzale Aldo Moro 5, 00185 Roma, Italy

[e]Networking Research Center on Bioengineering, Biomaterials and Nanomedicine (CIBER-BBN), Campus Universitari de Bellaterra, E-08193 Cerdanyola, Spain

* Email: luisa.giansanti@univaq.it; ventosa@icmab.es

S1. D_H and PDI (reported in bracket) of not dialysed quatsomes devoid of UA or containing UA added *i*) in the reactor during quatsomes formation or *ii*) on preformed liposomes by incubation. Reported D_H values were obtained one week after quatsomes preparation and correspond to the average values over at least three independent measurements. Reported errors correspond to mean of the standard deviation obtained by each independent measurement.

Formulation	without UA, nm	with UA, nm (reactor)	with UA, nm (incubation)
chol/CS **12**	99±3 (0.17)	110±3 (0.19)	120±2 (0.15)
chol/CS **14**	79±4 (0.19)	82±4 (0.24)	112±1 (0.16)
chol/CS **16**	63±2 (0.23)	55±2 (0.18)	51±3 (0.23)
chol/N-ox **12**	≈1000 (0.63)	≈1000 (0.54)	≈1000 (0.56)
chol/N-ox **14**	83±2 (0.16)	80±2 (0.18)	109±3 (0.12)
chol/N-ox **16**	80±4 (0.23)	191±4 (0.31)	180±4 (0.38)

S2. D_H and PDI (reported in bracket) of not dialysed quatsomes devoid of UA or containing UA added *i*) in the reactor during quatsomes formation or *ii*) on preformed liposomes by incubation. Reported D_H values were obtained 4 months after quatsomes preparation and correspond to the

average values over at least three independent measurements. Reported errors correspond to mean of the standard deviation obtained by each independent measurement.

Formulation	without UA, nm	with UA, nm (reactor)	with UA, nm (incubation)
chol/CS **12**	127±5 (0.17)	144±3 (0.18)	148±4 (0.16)
chol/CS **14**	112±4 (0.19)	112±4 (0.26)	140±5 (0.18)
chol/CS **16**	67±2 (0.19)	60±2 (0.20)	55±3 (0.21)
chol/N-ox **12**	> 1000, polydisperse	> 1000, polydisperse	> 1000, polydisperse
chol/N-ox **14**	115±4 (0.22)	105±3 (0.18)	138±6 (0.15)
chol/N-ox **16**	85±4 (0.25)	185±5 (0.29)	177±3 (0.31)

S3. ζ-potential of the investigated quatsomes with and without UA in water. All values reported were obtained by the average of 3 consecutive measurements of the same samples.

Formulation	without UA, mV	with UA, mV (reactor)	with UA, mV (incubation)
chol/CS **12**	+49±2	+55±2	+51±1
chol/CS **14**	+78±3	+73±3	+73±1
chol/CS **16**	+90±1	+86±1	+91±6
chol/N-ox **12**	-40±2	-35±2	-38±2

chol/N-ox **14**	+73±2	+69±2	+65±3
chol/N-ox **16**	+69±3	+81±3	+83±1

S4. Entrapment Efficiency (E.E., percentage) of UA loaded in the investigated quatsomes.

Formulation	E.E. of UA in the reactor[a]	E.E. of incubated UA[b]
chol/CS **12**	45±4	99±2
chol/CS **14**	80±3	100±5
chol/CS **16**	96±5	89±3
chol/N-ox **12**	100±4	92±1
chol/N-ox **14**	100±2	100±3
chol/N-ox **16**	98±3	91±2

[a]UA was added directly in the reactor during Quatsomes preparation;[b] UA was included in preformed Quatsomes upon incubation

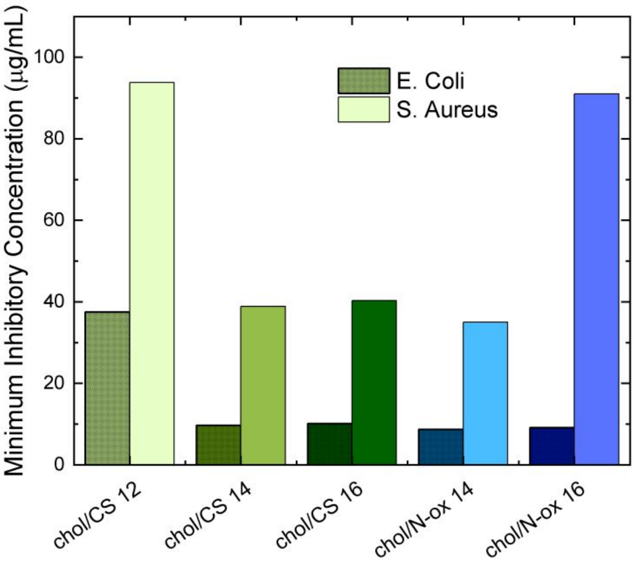

S5A. Minimum inhibitory concentration (MIC) of the different Quatsome formulations on Methicillin resistant *Staphylococcus aureus* and *Escherichia* ~~E~~*coli* bacterial strains (in µg/mL of total lipid concentrations).

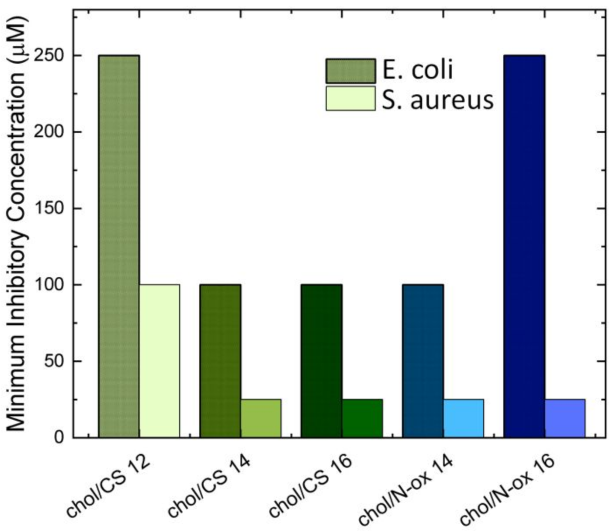

Figure S5B. Minimum inhibitory concentration (MIC) of the different Quatsome formulations on Methicillin resistant *S. aureus* and *E. coli* bacterial strains (molarity of components of Quatsomes).
